# THE BUDDY PROGRAM: IMPACT ON PERSONS LIVING WITH DEMENTIA AND FAMILY CAREGIVERS

**DOI:** 10.1093/geroni/igz038.1528

**Published:** 2019-11-08

**Authors:** Darby Morhardt, Mary Mittelman, Ann Burgunder, Thea Micoli

**Affiliations:** 1 Northwestern University, Chicago, Illinois, United States; 2 New York University, New York, New York, United States

## Abstract

Psychosocial interventions have the potential to offer substantial benefit to people with dementia and their family caregivers. The Buddy Program is an experiential learning program that pairs students with persons with dementia for activities and relationship-building. Previous studies have demonstrated the program’s positive impact on student knowledge and attitudes. New York University’s (NYU) Alzheimer’s Family Support Program began replicating the Buddy Program in 2017 and has enrolled 80 students. Northwestern University’s (NU) Buddy Program, in its 22nd year, has enrolled 260. This presentation describes the impact of the program on the mentors (NU) and the caregivers (NYU). Post program focus groups with mentors and student journals describing interactions with the caregiver were thematically analyzed. Mentors describe feelings of pride in the mentorship role, enjoyment being with student, and the opportunity to develop a new friendship. Caregivers enjoyed the respite provided by the buddies, knowing that their relatives with dementia were enjoying themselves and seeing their relatives with dementia in the role of valued companion. Qualitative data from caregivers and mentors participating in the buddy programs at NU and NYU underscore the possibility that people with dementia can still make contributions to society. The buddy program has a positive impact on quality of life for persons living with dementia and their family members, while providing a valuable educational experience for students. Further research on the buddy program’s benefits is warranted.

